# Clinicians’ perceptions for indicating and contra-indicating integrated treatment for SUD and comorbid PTSD, a vignette study

**DOI:** 10.1186/s13011-019-0194-5

**Published:** 2019-02-19

**Authors:** Germa Catherina Maria Nass, Leon Willem van Rens, Boukje Arnolda Gerdina Dijkstra

**Affiliations:** 1 IrisZorg Addiction Treatment, P.O. Box 351, 6800 AJ Arnhem, The Netherlands; 2 NISPA, Nijmegen Institute for Scientist-Practitioners in Addiction, Radboud University, P.O. Box 6909, 6503 GK Nijmegen, The Netherlands

**Keywords:** Substance use disorder, Posttraumatic stress disorder, Integrated treatment, Vignette study, Comorbidity

## Abstract

**Background:**

Posttraumatic Stress Disorder (PTSD) is more common in patients with Substance Use Disorder (SUD) than in the general population. Although international guidelines recommend integrated treatment clinicians are still hesitant in offering integrated treatment and more concrete recommendations are needed. This study aims to contribute to a practice-based guideline through the exploration of practice-based decision criteria to determine the indication and treatment of SUD and PTSD.

**Methods:**

A vignette study to explore the views of experienced clinicians on the treatment of SUD and PTSD.

**Results:**

Thirty-one experienced clinicians working in Dutch addiction care facilities filled in 15 vignettes resulting in 465 scored vignettes. Respondents did not report any contra-indications for integrated treatment and the perceived relationship between SUD and PTSD was found to be an important factor in the indication of integrated treatment.

**Conclusions:**

For integrated treatment to be offered to all eligible patients more training and schooling in trauma treatment and comorbid psychopathology is needed for all disciplines involved. Inpatient treatment options are necessary when patients need external support due to psychiatric or physical vulnerabilities. Further research into the effect of the relationship between SUD and PTSD on treatment execution and effectiveness is needed and can contribute to future treatment guidelines.

## Background

The comorbidity of Substance Use Disorder (SUD) and Posttraumatic Stress Disorder (PTSD) is common and internationally reported prevalence rates vary from 15% [1] up to 52% [2]. In the Netherlands 17% [3] to 37% [4] of patients entering an inpatient or outpatient addiction treatment program have SUD and lifetime PTSD. For residential SUD treatment percentages are considerably higher, e.g. 46.2% [5]. It is clear that PTSD is more commonly found in patients with substance use disorders than in the general population, in which it is about 10% [4, 6].

There are several international guidelines that recommend an integrated treatment of both disorders [7–10] because of indicated better outcome than treatment in which the disorders are treated separately [8–11]. Patients also prefer a simultaneous, integrated treatment for both disorders [12, 13]. In practice, however, patients often report separate treatment for each disorder. This is supported by work completed by Najavitis [14], in which clinicians rated integrated treatment of dual diagnosis (SUD and PTSD) as more difficult to treat than either disorder alone. Despite the guideline recommendations and patient preferences PTSD seems greatly undertreated in patients with SUD [8, 15–18].

Therapists working in addiction care are hesitant to use an integrated treatment because they are afraid that trauma-focused treatment will be harmful, counterproductive or that it will form a distraction from SUD treatment [15]. Blakey and Bowers [19] found that professionals in SUD treatment believed that an integrated approach would take more time than was allocated to them, and reported a lack of knowledge and absence of qualified professionals. Therapists also had strong beliefs that addiction must be treated first because they viewed addiction to be the primary disorder and thought abstinence a prerequisite for psychiatric treatment. Furthermore, the historical separation between substance abuse treatment and mental health systems (with each system predominately focusing on just one disorder) does not facilitate an integrated treatment approach [19].

Research results on the topic of contra-indications are somewhat inconsistent, giving little concrete support to clinicians seeking guidance in treating this complex dual diagnosis. In their review, Van Minnen et al. [20] conclude that substance use disorder is not a contra-indication for trauma focused PTSD treatment. Mills et al. [7] found that patients who received exposure treatment for PTSD did not demonstrate poorer substance use outcomes compared to patients who received SUD treatment alone. However a more recent review [21] suggests that there may be problems with tolerability of trauma-focused intervention for some patients with SUD and PTSD due to more drop-out in patients receiving trauma-focused interventions.

Guidelines are not explicit in which patients should be excluded from integrated treatment. It is possible that lack of clarity when it comes to contra-indications makes clinicians overly careful and reserved in the administration of integrated treatment. Research into integrated therapy for patients with trauma and substance dependency often exclude the following comorbidities: psychotic symptoms, suicidal ideations and severe cognitive impairment [8, 22–26]. Recent research indicates that psychotic symptoms need not be a contra-indication for the treatment of PTSD [27]. In addition to the psychiatric factors that may be viewed as contra-indications there are contextual factors that are often considered important preconditions. Safety, day structure, social support and stabilisation of substance use are believed to be essential preconditions for trauma therapy [28, 29]. Hospitalization or inpatient treatment can sometimes offer more safety, structure and support than can be reached in outpatient treatment programs. However most research to date has been done on outpatient populations. Markus et al. [30] advise an outpatient setting where possible and an inpatient setting when needed, but does not specify decision criteria. Another reason for hesitancy in therapists is that they struggle with questions of when to initiate trauma interventions and how to cope with on-going substance use [12].

Recent research by (van Rens LW, Gielen N, Nass GCM, Dijkstra BAG: Implementation of integrated treatment for comorbid substance use disorder and posttraumatic stress disorder: a survey among clinicians in Dutch addiction care, submitted) confirms the need for concrete guidelines with respect to contra-indications from the perspective of clinicians working in Dutch addiction care. Although their view toward integrated treatment is generally positive, it appears clinicians need more guidance in the process of indicating or contra-indicating PTSD treatment during addiction treatment.

The present study uses patient vignettes to further explore the views of experienced clinicians on the treatment of SUD and PTSD in more depth resulting in practice-based decision criteria to guide the indication of integrated treatment of SUD and PTSD. Vignettes are often used to identify decision making processes of medical and paramedical professionals with respect to diagnostics, needs assessment and treatment [31, 32]. The use of vignettes makes it possible to explore clinician’s views accurately and realistically and reduces socially desirable responses. Our main questions are 1) Which psychiatric and contextual factors are taken into account by experienced clinicians working in addiction care when deciding if a patient should receive integrated treatment for SUD and PTSD? and 2) Which factors are taken into account when indicating treatment setting? The results of this study provide guidance for clinicians in the process of indicating and contraindicating integrated treatment, and in the execution of integrated treatment.

## Methods

### Design and participants

A prospective vignette study was conducted among healthcare and clinical psychologists working in addiction care facilities in the Netherlands. Psychologists were first approached in April and May of 2016 and again in June and August of 2016 if there was no response. The study protocol was approved by the institute’s internal scientific committee. Participants participated in the study voluntarily and they were guaranteed anonymity.

### Procedures

#### Construction of vignettes

Psychologists working in addiction care facilities were asked to provide researchers with patient cases. The anonymized cases had to be based on a real patient with at least a PTSD and SUD classification on the DSM-IV. Psychologists were asked to state patient gender, all psychiatric classifications according to the DSM-IV, actual psychiatric state, previous psychiatric and/or addiction treatment and the actual situation with respect to housing, work and relationships.

Psychologists provided 20 patient cases. Five cases were excluded: one had no PTSD classification, the other four were excluded due to overlap with other cases. The remaining 15 cases were then poured into a similar format, called the vignettes. To ensure anonymity the cases were altered. Yet alterations were kept to a minimum to keep the vignettes realistic. The vignettes represent the wide array of substances used by patients in Dutch addiction care (e.g. alcohol (*n* = 10), cannabis (*n* = 7), opiates (*n* = 1), amphetamines (*n* = 1), cocaine (*n* = 2) and GHB (*n* = 2)) and the range in severity of symptoms associated with PTSD. They also include the variety of comorbid symptoms and disorders often seen in this population, like personality disorders (borderline and antisocial features in particular), depression, psychotic symptoms, intellectual disabilities, suicidal tendencies and cognitive dysfunctions. A short description of the vignettes is found in Table 2.

#### Recruitment of respondents

Healthcare and clinical psychologists from the Van Rens study (van Rens LW, Gielen N, Nass GCM, Dijkstra BAG: Implementation of integrated treatment for comorbid substance use disorder and posttraumatic stress disorder: a survey among clinicians in Dutch addiction care, submitted) who indicated that they were willing to take part in further research, were approached via e-mail. Additionally, other psychologists working in Dutch addiction care were approached through snow-ball sampling. In snow-ball sampling the researcher asks an already approached respondent to point out possible new respondents. The new respondents were then also approached via e-mail and asked to point out possible new respondents. Non-responders were sent a reminder mail. After this, non-responders were asked one more time to complete the survey or to reply that they were not willing or able to participate.

Of the 67 clinicians that were approached via mail three indicated they had insufficient time to complete the survey. One clinician indicated insufficient experience pertaining to the patient population and 29 did not respond. Three were excluded afterwards due to insufficient work experience with this population (less than 2 years). Participants were informed twice via e-mail and via the questionnaire about the goal of the study, the extent of time and effort required, the anonymity of results and the voluntary nature of participation. Thirty-one participants (a response rate of 46.3%) filled in 15 vignettes which resulted in 465 scored vignettes with a possible maximum of three open answers (total maximum of 1395). The average age of our sample of clinicians working in four different addiction care facilities was 40.1 years (SD 8.6) with an average of 7.2 years (SD 4.6) of work experience in PTSD treatment. The clinicians were mostly healthcare psychologists (87.1%), ten of which were in training to become specialists. Further participant characteristics are summarized in Table 1.

**Table 1:** Demographic data of participating clinicians (*n*=31)

Age, mean (sd)	40.1 (8.6)
Work experience PTSD, mean (sd)	7.2 (4.6)
Profession, n (%)^a^	
Psychologist	1 (3.2)
Health-care psychologist	27 (87.1)
Psychotherapist	3 (9.7)
Clinical psychologist	2 (6.5)
Clinical child, family and education studies	1 (3.2)
Level of care, n (%)^b^	
Outpatient	21 (67.7)
Residential treatment	12 (38.7)
Day treatment	1 (3.2)

^a^3 participants are psychotherapists as well as health care psychologists

^b^1 participant works in all of the three level of care settings and 2 participants work in a residential treatment setting as well as in an outpatient treatment setting

#### Online survey vignettes: treatment recommendations

Respondents were issued a personal link to an online survey containing the vignettes. They were asked A) to which extent they would recommend integrated treatment for SUD and PTSD for each vignette by grading their decision on a visual analogue scale (VAS) with a quantitative score from − 5 to + 5. A score of − 5 meant that they were absolutely certain that they would not indicate integrated treatment and + 5 meant that they were absolutely certain that they would indicate integrated treatment for the patient in that vignette. Respondents were then invited to B) give three reasons for their decision. Following this they were asked which intervention they would administer (detoxification, EMDR, stabilization, cognitive therapy, exposure (imaginal and/or in vivo and/or writing therapy and/or rescripting), medicinal treatment, other intervention). These interventions were based on national and international evidence-based guidelines for treatment of PTSD and SUD [9, 33]. Respondents were requested to take the most ideal situation in mind when making their treatment indication decisions. That is independently of the possibilities of the respondents’ workplace or the institution they work for.

Correspondingly respondents indicated C) whether they would designate an outpatient or an inpatient setting for each vignette. Again three reasons for this decision could be given (D). For the first five vignettes they also specified E) impeding or facilitating factors for the treatment that they recommended in their own place of work. In contrast to step A to D, step E is about the realistic scenario (e.g. own resources and expertise) instead of the most ideal situation.

#### Determination of decision criteria

Based on the derived decision criteria (for further details see data analysis), we formulated recommended criteria for the indication of integrated treatment.

### Data analysis

Data was processed and analysed using the Statistical Package for the Social Sciences (SPSS), version 22. A descriptive analysis of the respondents VAS score was carried out per vignette. The percentage (frequency) of the respondents scores within the range ≥ + 3 to + 5 (integrated treatment of SUD and PTSD) or ≤ − 3 to − 5 (no integrated treatment) was calculated for each vignette. A percentage of 60 or higher within this range was considered to indicate an adequate level of consensus between the respondents. To obtain the decision criteria for the indication of integrated treatment interpretive recursive abstraction qualitative analysis [34] was used by two research psychologists (authors GN and LvR). The three reasons given by all clinicians for and against the indication of integrated treatment were coded by each research psychologist independently. After consensus was reached 30 categories were determined. These were further discussed and reduced to 25 categories. Following this the categories were divided into three factors: psychiatric factors, contextual factors and substance use factors. The decision criteria for in- or outpatient treatment were obtained in the same way, as were impeding and facilitating factors given by clinicians in the first five vignettes.

## Results

### Analysis of vignettes

The mean scores, standard deviation (SD) and range of scores are shown per question for each vignette (Tables 2 and 3). Some vignettes showed that clinicians had opposite choices, with scores varying between − 5 and + 5. In most vignettes the SD was relatively high, indicating that the mean and SD were only partly informative. For this reason the frequency scores were used.

**Table 2:** Descriptive results per vignette scored by clinicians (*n* = 31) on the indication of integrated treatment of SUD and PTSD

Vignette	min	max	mean (SD)	% ≤ − 3 no integrated treatment	% ≥ + 3 integrated treatment	% ≥ + 3 after filter non- choosers (n)
1. Female, 45, cocaine, heroin, cannabis. Multiple traumatic events (sexual abuse, rape and violence) and prostitution. Complex PTSD and BPD with antisocial tendencies. Many social and contextual problems.	−4	5	3.2 (2.0)	3.2	**71.1**	**73.3** (30)
2. Male, 33, alcohol and cannabis. Abused for many years by male babysitter. PTSD and BPD, followed DGT. Has residence and a job. In debt, has a limited social network	−3	5	2.8 (2.3)	3.2	64.6	69.0 (29)
3. Male, 23, excessive amounts of alcohol each weekend leading to amnesia after seeing his friend fall to his death. Re-experiences the accident, feels guilty. Lives with his girlfriend, has a job.	−4	5	3.3 (2.5)	6.4	**77.4**	**82.8** (29)
4. Male, 59, alcohol, many clinical therapies. Periods of sobriety. PTSD, physical and sexual abuse, intellectual disability. On sick leave, drinks after half past five. Suicidal tendencies 3 weeks ago. Girlfriend, acquaintances drink.	0	5	3.8 (1.4)	0.0	**77.5**	**80.0** (30)
5. Female, 25, cannabis, alcohol and GHB. Personality disorder NOS, PTSD. Stepfather was violent and stepbrother was sexually inappropriate. Removed from home at young age. Many different institutions and addresses. Several suicide attempts, now anxiety, depression and trouble controlling emotions.	0	5	3.8 (1.7)	0.0	**77.5**	**85.7** (28)
6. Female 55, social drinker until an armed robbery. Since then 2 bottles of wine a day to lower anxiety and tension caused by the trauma. Married, two daughters, works, exercises and makes music.	−3	5	3.6 (2.0)	3.3	**74.3**	**82.1** (28)
7. Female, 61, alcohol and PTSD. Has a daughter from her father who abused her and gave her alcohol. Daughter given up for adoption contacted patient 5 years ago and alcohol consumption further increased. Partner and 2 adult children.	0	5	3.1 (1.6)	0.0	**71.0**	**81.5** (27)
8. Female, 32, cannabis, abstinent for 5 months after treatment, chronic PTSD. Labelled ‘untreatable’ during childhood. Despondency and negative self-image. No work, friend abuse drugs.	−5	5	0.7 (3.5)	29.0	38.7	44.4 (27)
9. Female, 20, cannabis and cocaine, many traumatic experiences. Symptoms consistent with PTSD and indications found for impaired personality development, probably Clusters B (problems with regulation of emotions, fragile self-image). No permanent address, no income.	−4	5	3.0 (2.5)	6.4	**74.2**	**82.1** (28)
10. Female, 30, Turkish, cannabis dependency in remission after treatment. PTSD and depression. Lives with sister, no social network. Suicidal in the past.	−4	5	1.1 (3.1)	16.2	35.5	39.3 (28)
11. Female, 31, GHB, complex PTSD and psychotic disorder NOS. Exhibits borderline personality traits during long-term GHB use. Hospitalized several times for GHB detox. Has her own home. Parents are supportive, are her children’s guardians. Partner is supportive, but also uses GHB.	−4	5	2.7 (2.4)	3.2	**61.4**	**76.0** (25)
12. Male, 20, Moroccan, alcohol, depressive symptoms, PTSD, latent suicidality and borderline intellectual functioning. Homosexual orientation which his culture does not accept and parents do not know about. Physically abused by a group because of his sexuality. Lives with residential guidance, dept., no daytime activities.	−4	5	2.9 (2.0)	3.2	**67.7**	**75.0** (28)
13. Female, 52, alcohol and amphetamine, PTSD, psychotic disorder NOS and cognitive disorders due to physical disorder. Is abstinent. Lives independently with guidance, works on a care farm.	−4	5	2.3 (2.4)	6.4	58.0	**66.7** (27)
14. Female, 66, alcohol use halved with Campral. Drinks since her ‘coming-out’ as a lesbian. Happy with her life. Suffers form anxiety and avoidance after a car accident 10 years ago. Lives with partner, on sick leave from her job. Fairly extensive social network.	−5	5	2.1 (2.9)	9.6	58.1	**62.1** (29)
15. Male, 20, alcohol and cannabis. History of amphetamine dependency. ADHD, stagnated emotional development and PTSD. Mandatory contact with addiction care because of joyriding under the influence. Insecure attachment in his upbringing and bullying, fundamentally insecure. Lives in a sheltered housing project, has a partner.	−3	5	3.3 (1.8)	3.2	**74.2**	**79.3** (29)

N.B. Numbers in bold indicate that the percentage of scores ≥ + 3 or ≤ − 3 was ≥60% (considering an adequate level of consensus between the respondents)

**Table 3:** Descriptive results per vignette for in- or outpatient treatment scored by clinicians who were part of the consensus group that indicated integrated treatment (score of ≥ + 3 on question 1, *n* = 13)

Vignette	N clinicians	min	max	mean (SD)	≤ − 3%^a^ outpatient setting	% ≤ − 3 after filter non choosers (n)	≥ + 3%^a^ inpatient setting	% ≥ + 3 after filter non choosers (n)
1	23	−3	5	1.4 (2.8)	13.0	14.6 (22)	47.8	50.0 (22)
2	20	−5	5	−2.8 (2.4)	**70.0**	**77.8** (18)	4.8	5.6 (18)
3	24	−5	3	−3.6 (2.1)	**70.9**	**77.3** (22)	4.2	4.5 (22)
4	24	0	5	3.4 (1.3)	0.0	0.0 (23)	**79.1**	**82.6** (23)
5	25	−3	5	4.0 (1.8)	4.0	4.0 (25)	**88.0**	**88.0** (25)
6	23	−5	0	−4.1 (1.2)	**91.3**	**95.4** (22)	0.0	0.0 (22)
7	22	−5	4	−0.9 (3.0)	36.3	53.3 (15)	13.6	20.0 (15)
9	23	−5	5	2.4 (2.8)	8.6	10.0 (20)	**69.5**	**80.0** (20)
11	19	−5	5	1.9 (2.5)	5.3	6.2 (16)	42.1	50.0 (16)
12	21	−5	4	−1.4 (3.1)	38.0	42.1 (19)	14.3	15.8 (19)
13	18	−5	5	−1.8 (3.5)	50.0	52.9 (17)	22.3	23.5 (17)
14	18	−5	4	−3.1 (2.5)	**77.8**	**93.3** (15)	5.6	6.7 (15)
15	23	−5	2	−2.5 (2.0)	**60.9**	**70.0** (20)	0.0	0.0 (20)

^a^ Frequency percentage score per vignette: outpatient setting (from ≤ − 3 to −5) or inpatient setting (from ≥ + 3 to + 5)

N.B. Numbers in bold indicate that the percentage of scores ≥ + 3 or ≤ − 3 was ≥60% per vignette (considering an adequate level of consensus between the respondents)

#### Integrated treatment or not

For the choice of whether to recommend integrated treatment or not 11 of the 15 vignettes had an adequate level of consensus between the respondents (Table 2). Deleting clinicians that made no indication choice (score 0) results in two extra vignettes (13 and 14) with an adequate level of consensus. In all of the consensus vignettes clinicians recommended integrated treatment.

#### Decisive criteria for integrated treatment or not

The most commonly given reasons per consensus vignette are stated in Table 4. The interrelatedness of SUD and PTSD is the reason most often given for the indication of integrated treatment followed by the reason that PTSD maintains SUD. The most often stated reason for not recommending integrated treatment of SUD and PTSD is the severity of SUD (i.e. substance use was in remission).

**Table 4:** Reasons given by clinicians for their recommendation of integrated treatment in consensus vignettes (*n* = 13)

Vignette	No of clinicians with score + 1 to + 5 for integrated treatment	Most commonly cited reasons for integrated treatment
1	28	PTSD maintains SUD (17), interrelatedness PTSD and SUD (9)
2	25	Interrelatedness PTSD and SUD (10), PTSD maintains SUD (9), patient wishes (7), limited SUD (6)
3	26	Interrelatedness PTSD and SUD (11), PTSD maintains SUD (9)
4	30	Interrelatedness PTSD and SUD (15), PTSD maintains SUD (8), patient wishes (6)
5	27	Interrelatedness PTSD and SUD (12), PTSD maintains SUD (6), complexity of PTSD (6), complexity of co-morbid psychopathology (5)
6	26	Interrelatedness PTSD and SUD (13), PTSD maintains SUD (6), patient wishes (6)
7	26	Interrelatedness PTSD and SUD (15), PTSD maintains SUD (5), long duration of SUD (4)
9	24	PTSD maintains SUD (9), interrelatedness PTSD and SUD (7)
11	23	Interrelatedness PTSD and SUD (6), severity of SUD (5), complexity of co-morbid psychopathology (5)
12	26	Interrelatedness PTSD and SUD (8), PTSD maintains SUD (4), complexity of co-morbid psychopathology, single-event trauma (4)
13	23	Interrelatedness PTSD and SUD (5), severity of SUD (5), level of suffering (4), PTSD maintains SUD (3), complexity of co-morbid psychopathology (3)
14	23	severity of SUD (6), Interrelatedness PTSD and SUD (5), patient wishes (4), PTSD maintains SUD (4)
15	27	Interrelatedness PTSD and SUD (10), PTSD maintains SUD (7), complexity of co-morbid psychopathology (5), patient wishes (4)

#### Outpatient or inpatient setting

In three of 13 vignettes with a consensus for integrated treatment clinicians were collectively convinced that inpatient treatment was indicated. Outpatient treatment was indicated for five vignettes. Deleting clinicians that made no choice did not influence the results (Table 3).

#### Decision criteria for outpatient or inpatient setting

Table 5 shows the most commonly given reasons for the eight vignettes in which there was an adequate level of consensus, such as daytime activities and adequate support system for outpatient treatment; lack of support system, suicide risk and susceptibility to crisis for inpatient treatment.

**Table 5:** Reasons given by clinicians for their choice of treatment setting for vignettes in which there was consensus as to integrated treatment and treatment setting (*n* = 8)

Vignette	No of clinicians	Choice of setting	Most commonly cited reasons (n)
2	17	Outpatient	Daytime activities (13), patient stability (8), adequate support system (7), limited SUD (6), earlier treatment (5)
3	21	Outpatient	Daytime activities (12), limited SUD (11), adequate support system (6)
4	23	Inpatient	Suicide risk (14), severity of SUD (13), lack of support system (13)
5	24	Inpatient	Drug of choice (13), suicide risk (11), vulnerability to psychosis (6), susceptibility to crisis (6), lack of support system (5)
6	22	Outpatient	Adequate support system (15), daytime activities (12), limited SUD (5), patient stability (5)
9	17	Inpatient	Housing situation (15), susceptibility to crises (9), lack of support system (4)
14	14	Outpatient	Adequate support system (11), single-event trauma (5), limited SUD (5)
15	18	Outpatient	Adequate support system (17), housing situation (3)

#### Impeding or facilitating factors

The most frequently stated impeding factor for executing integrated treatment in their own place of work was insufficient expertise. In many answers this pertained to a lack of expertise in integrated treatment of SUD and PTSD available in the work setting. However, insufficient expertise in other comorbidities such as personality disorders and cognitive impairment was also mentioned. Not having the possibility to provide inpatient treatment was also found to be an impeding factor as was not having the possibility to offer (time) intensive treatment. The most frequently mentioned facilitating factor was the presence of sufficient expertise in the treatment of PTSD.

### Determination of decision criteria

Based on the results the following recommended criteria for the indication of integrated treatment could be formulated: the interrelatedness of SUD and PTSD, PTSD maintains SUD, patients’ wishes, and the complexity of comorbid psychopathology. For choice of setting the recommended criteria could be divided into three categories, namely psychiatric factors, contextual factors and substance use factors. The criteria are shown in Table 6.

**Table 6:** Recommended criteria for indication of in- or outpatient setting for the integrated treatment of SUD and PTSD

Criteria outpatient	Criteria inpatient
**Substance abuse**	**Substance abuse**
Limited SUD	Severity of SUD
	Drug of choice
**Psychiatric**	**Psychiatric**
Patient stability	Suicide risk
Earlier treatment	Vulnerability to psychosis
Single-event trauma	Susceptibility to crisis
**Contextual**	**Contextual**
Daytime activities	Lack of support system
Adequate support system	Inadequate Housing situation
Stable Housing situation	

## Discussion

This prospective vignette study was conducted in order to contribute to a practice-based guideline in the treatment of comorbid SUD and PTSD. The aim was to determine practice-based decision criteria in the indication of integrated treatment for patients with comorbid SUD and PTSD and in the indication of treatment setting (in or outpatient) when integrated treatment is indicated.

In 86% of the presented cases an adequate level of consensus was reached and integrated treatment was recommended. The relationship between SUD and PTSD was found to be the most important decision criterion for the indication of integrated treatment. The decision criteria for treatment setting, underlined the necessity of stability of a patients’ psychiatric, SUD and contextual (social and living) situation. Outpatient treatment is preferred when there is stability in daily activities, housing and comorbid psychopathology. Inpatient treatment appears to be indicated when patients need external support due to vulnerability. This can include vulnerability as a result of comorbid psychopathology, drug of choice (particularly GHB use), but also due to lack of a support system. The two most often mentioned impeding factors for executing integrated treatment in their own place of work were the absence of inpatient treatment possibilities and lack of expertise. In the non-consensus vignettes substance use was already in remission. This may explain the lack of consensus: some clinicians found SUD sufficiently treated and chose to focus on PTSD-treatment alone, while other clinicians advised continuation of treatment of both SUD and PTSD. We do not consider this as a contra-indication for integrated treatment, but as the absence of indication for integrated treatment.

The use of vignettes in this study made it possible to explore the views of experienced clinicians more realistically than the use of questionnaires. Vignettes reduce socially desirable responses and reflect clinical complexity [35]. In our study we specifically asked experienced clinicians to take the most ideal situation in mind, independent from their workplace possibilities. We found that a vast majority of respondents preferred integrated treatment of SUD and PTSD. Noteworthy is that the present study found the underlying reason for this preference to be the clinicians’ opinion that PTSD maintains SUD. This is in line with research findings [36, 37] which suggest that PTSD patients use drugs to self-medicate for PTSD symptoms. Brown and Wolfe [38] pose that the development of SUD may be a function of the stressor experienced. An alternative explanation for the relationship between SUD and PTSD is that problematic substance use increases the risk of exposure to traumatic events, but research findings are inconsistent [39]. Besides these causal models the shared liability model proposes that both disorders share common risk factors such as genetic risk and personality traits [2, 39]. Patients often perceive the two disorders to be functionally related and prefer integrated treatment [15].

Strikingly, in our study experienced clinicians did not report contra-indications for integrated treatment, even in vignettes in which there was high problem complexity and complex comorbid psychopathology. This is in contrast to results found in studies by Becker et al. [40] and Hagenaars et al. [41] in which clinicians found depression, suicidality, dissociation, psychosis and comorbid anxiety disorders to be contra-indications. Our findings may be the result of our instruction to keep an ideal situation in mind, but also the use of vignettes makes it more realistic. Problem complexity and complexity of comorbid psychopathology were only found to be important with respect to deciding treatment setting and not for deciding integrated treatment. From this, it is not surprising that a lack of inpatient treatment possibilities was a frequently mentioned impeding factor for executing integrated treatment in their own place of work. The absence of clinical beds can result in a more reserved approach in the indication of integrated treatment of SUD and PTDS. Besides the possibility of inpatient treatment, clinicians also stress the importance of appropriate schooling and training for therapists and other disciplines in both trauma treatment and comorbid psychopathology, both in inpatient and outpatient settings. Clinicians need to be able to intensify outpatient support or outpatient treatment if necessary. This requires a sufficient amount of care workers and therapists with adequate expertise. A perceived lack of expertise can result in reluctance to indicate integrated treatment.

One limitation of the present study is that the recruitment procedure may have contributed to selection bias. Clinicians interested in integrated treatment or with a strong belief in integrated treatment may have been more inclined to take part in the study. However this was the target group we wanted to include because of their expertise on the subject. Also respondents were all working in Dutch addiction care which limits generalization beyond addiction care and beyond Dutch borders. EMDR, for example is an intervention in which most psychologists working in the Netherlands are trained. These Dutch therapists often feel better equipped to treat PTSD with EMDR than with exposure. This may however not be the case in other countries. As written before we would like to emphasize that *indication* for integrated treatment, as reported by the respondents, does not reliably reflect the actual *usage* of integrated treatment. A merit of the study is the use of a large number of vignettes which made it possible to generate a large response. Furthermore the vignettes were well constructed as we found psychiatric, addiction and contextual factors in the reasons given. A replica study in a sample of non-experienced clinicians to investigate whether they recommend integrated treatment or not is recommended, the same applies for a replica study in PTSD setting.

Present results emphasize the importance of appropriate training and education for therapists administrating integrated treatment and for other disciplines involved. Furthermore the support and safety-net of inpatient treatment possibilities can help clinicians in their decision to offer more vulnerable patients the guideline advised treatment for SUD and PTSD. Our findings stress the importance of future studies on the relationship between SUD and PTSD. It is not unthinkable that integrated treatment may be more effective in patients who have a strong conviction that both disorders are related. Current guidelines recommend integrated treatment, but some research indicates that treatment effects vary [21, 24]. Research into the effects of patients’ beliefs or clinicians’ opinions about interrelatedness on the (outcome of) treatment of PTSD in patients with SUD can help to explain different treatment effects and offer further contribution to practice based guidelines.

## Conclusion

In conclusion, experienced clinicians did not report contra-indications for integrated treatment of SUD and PTSD, so integrated treatment should be offered when comorbid SUD and PTSD is present. When integrated treatment was not indicated substance use was already in remission. We do not consider this as a contra-indication for integrated treatment, but as the absence of indication for integrated treatment.

The perceived relationship between SUD and PTSD is an important factor in the administration of integrated treatment. Further research into the relationship between SUD and PTSD and its effect on treatment execution and treatment results is needed and can contribute to future treatment guidelines. Inpatient treatment should be considered when patients need external support due to psychiatric and/or physical vulnerabilities. For integrated treatment to be offered to all eligible patients however more training and sufficient inpatient treatment options are necessary.

## Abbreviations

PTSD: Posttraumatic Stress Disorder
SUD: Substance Use Disorder
